# Maternal Systemic Inflammation and Fetal Thymic Size in Diabetic Pregnancies: Predictive Role of Hematological Biomarkers

**DOI:** 10.3390/jcm14217619

**Published:** 2025-10-27

**Authors:** Gülay Balkas, Şevki Çelen

**Affiliations:** Department of Perinatology, University of Health Sciences, Etlik Zübeyde Women’s Health Care Training and Research Hospital, 06010 Ankara, Turkey; sevkicelen@yahoo.com

**Keywords:** diabetes in pregnancy, fetal immune development, fetal thymus, obstetric ultrasound, systemic inflammatory biomarkers

## Abstract

**Background/Objectives:** This study aimed to evaluate the relationship between maternal systemic inflammatory indices, hematological parameters, and fetal thymus size, as measured by the thymus–thoracic ratio (TTR), among diabetic pregnancies, and to establish predictive cut-off values for reduced thymus size. **Methods:** This prospective cohort study enrolled 532 pregnant women, divided into four groups: pregestational diabetes mellitus (PGDM, *n* = 44), diet-controlled gestational diabetes mellitus (GDM, *n* = 73), insulin-treated GDM (*n* = 49), and normoglycemic controls (*n* = 366). Fetal thymus size, alongside serum levels of neutrophil-to-lymphocyte ratio (NLR), platelet-to-lymphocyte ratio (PLR), systemic immune-inflammation index (SII), systemic inflammation response index (SIRI), aggregate index of systemic inflammation (AISI), fibrinogen-to-albumin ratio (FAR), and C-reactive protein (CRP)-to-albumin ratio, were assessed in the third trimester. **Results:** All maternal diabetes subgroups demonstrated significantly reduced fetal thymus size compared with controls, with the most pronounced reduction observed in the PGDM group (*p* < 0.001). NLR, PLR, MLR, SIRI, AISI, and MPV were significantly elevated in the PGDM cohort, whereas CAR, FAR, and fibrinogen levels were markedly increased in the insulin-treated GDM group. Albumin levels were significantly decreased in both the PGDM and the insulin-treated GDM groups (*p* < 0.001). Among the evaluated biomarkers, AISI and FAR exhibited the highest diagnostic accuracy for predicting reduced fetal thymus size, with optimal cut-off values of 640.3 (sensitivity 82.3%, specificity 86.7%) and 0.114 (sensitivity 74.3%, specificity 88.7%), respectively. **Conclusions:** Maternal systemic inflammatory burden, as indicated by hematological biomarkers, is significantly associated with reduced fetal thymic size in diabetic pregnancies. These findings suggest that readily accessible blood-derived biomarkers, particularly AISI and FAR, may complement ultrasonographic evaluation, offering a cost-effective, non-invasive approach to predict compromised fetal immune development, especially in settings where direct thymic imaging is impractical.

## 1. Introduction

The escalating prevalence of impaired glucose metabolism during pregnancy constitutes a significant and growing global health challenge. Gestational diabetes mellitus (GDM), characterized by glucose intolerance first diagnosed during pregnancy, has a prevalence that varies according to diagnostic criteria, population genetics, and environmental factors [1,2,3]. In 2024, a study reported a global GDM prevalence of 14.7%, based on the International Association of Diabetes and Pregnancy Study Groups (IADPSG) criteria, which is the most widely used screening method worldwide [2]. Pregestational diabetes mellitus (PGDM), encompassing type 1 and type 2 diabetes diagnosed prior to conception, complicates 1–2% of pregnancies and is increasing in tandem with the global epidemic of obesity and insulin resistance among women of reproductive age [4,5].

Maternal dysglycemia is associated with an increased risk of adverse pregnancy outcomes, including congenital malformations, preeclampsia, polyhydramnios, spontaneous abortion, neonatal respiratory distress, stillbirth, and neonatal metabolic disturbances [6,7]. Beyond these immediate consequences, maternal hyperglycemia confers long-term risks of type 2 diabetes, metabolic syndrome, and cardiovascular disease for both mother and offspring [8,9], and is further linked to higher rates of respiratory, allergic, and neurodevelopmental disorders in offspring [10,11]. Emerging evidence additionally suggests that the impact of maternal dysglycemia transcends conventional metabolic risks, as heightened maternal inflammation may influence fetal immune ontogeny, thereby predisposing offspring to persistent immune dysregulation [12,13].

The fetal thymus serves as the primary site of T-lymphocyte maturation and differentiation, playing a pivotal role in establishing adaptive immunity [14]. Previous research has consistently demonstrated that adverse intrauterine conditions, such as chromosomal abnormalities, intrauterine infections, preeclampsia, and autoimmune diseases, can induce premature thymic involution [14,15]. Reduced thymic size is associated with compromised lymphoid development, heightened perinatal susceptibility to infection, and an increased long-term risk of immune dysregulation, encompassing autoimmunity and allergic disorders [15,16]. Ultrasound-based assessment of thymic dimensions, notably the thymus–thoracic ratio (TTR), has been established as a reproducible, gestational age–independent metric, facilitating non-invasive evaluation of fetal immune competence [17].

Normal pregnancy involves precisely orchestrated immunological adaptations to ensure tolerance of the semi-allogeneic fetus while maintaining robust antimicrobial defense [18]. This equilibrium is established through dynamic shifts between pro-inflammatory and anti-inflammatory states across gestation, mediated by meticulously regulated modulation of leukocyte subsets, cytokine signaling pathways, and acute-phase reactants [19]. Maternal hyperglycemia disrupts this balance, inducing chronic low-grade systemic inflammation characterized by heightened activity of neutrophils, monocytes, and activated platelets, as evidenced by elevated pro-inflammatory cytokines (e.g., IL-6), acute-phase reactants (e.g., CRP), and altered inflammatory biomarkers [20,21]. This inflammatory state may impair placental immune signaling, alter the intrauterine environment, and ultimately compromise fetal immune development, with the thymus identified as a critical target organ for these immunological disturbances [22,23,24,25,26].

In recent years, systemic inflammatory biomarkers—such as the neutrophil-to-lymphocyte ratio (NLR), platelet-to-lymphocyte ratio (PLR), monocyte-to-lymphocyte ratio (MLR), systemic immune-inflammation index (SII), systemic inflammatory response index (SIRI), aggregate index of systemic inflammation (AISI), fibrinogen-to-albumin ratio (FAR), and C-reactive protein (CRP)-to-albumin ratio—have garnered growing acceptance as cost-effective, readily accessible biomarkers of systemic inflammation [27,28,29]. Although extensively studied in the context of cardiovascular, oncologic, and metabolic disorders, their association with fetal immune parameters, particularly thymic size, remains insufficiently investigated.

We hypothesize that maternal hyperglycemia-induced inflammation, marked by elevated systemic inflammatory biomarkers, disrupts placental immune signaling and cytokine homeostasis, promoting fetal thymic involution through thymocyte apoptosis, oxidative stress, and impaired thymic epithelial maturation [23,24,25,26]. To the best of our knowledge, no prospective study has systematically investigated the relationship between maternal inflammatory and hematological biomarkers and fetal thymus size in pregnancies complicated by diabetes mellitus compared with normoglycemic controls. Elucidating such associations could yield novel, non-invasive insights into fetal immune development, thereby addressing a significant gap in perinatal medicine. Consequently, the primary objective of this investigation was to assess the association between maternal inflammatory indices, hematological parameters, and fetal thymus size, as determined by the TTR, during the third trimester of pregnancies affected by pregestational or gestational diabetes. A secondary objective was to establish cut-off values predictive of reduced thymus size, enabling the early identification of fetuses at increased risk of immune dysregulation.

## 2. Materials and Methods

### 2.1. Study Design

This prospective cohort study was conducted in accordance with the principles of the Declaration of Helsinki and received ethical approval from the local Ethics Committee from May to October 2022 (approval number: 05/30, dated 21 April 2022). Written informed consent was obtained from all participants prior to enrollment.

### 2.2. Study Population and Eligibility Criteria

A total of 718 pregnant women aged 18–40 years, attending routine antenatal outpatient visits between 20 and 40 weeks of gestation, were enrolled at the Perinatology Clinic of a tertiary care hospital. All participants underwent blood sampling during the same gestational window and subsequently delivered at the same institution.

**Figure 1:**
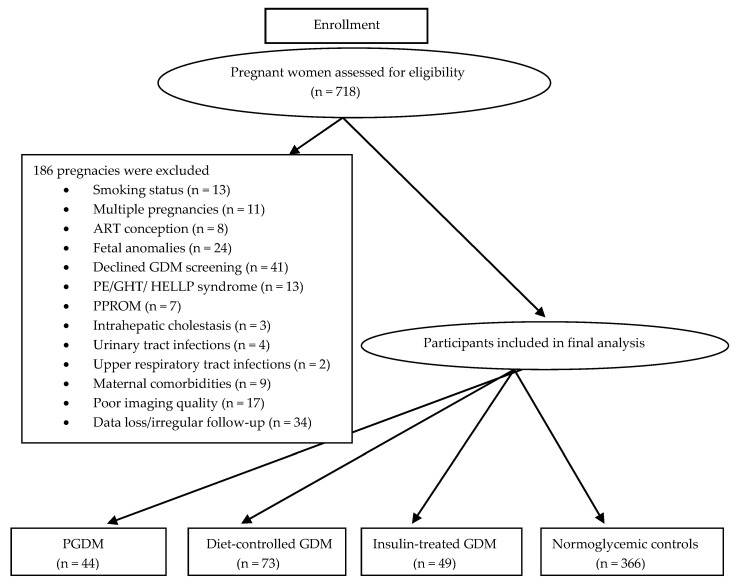
A flow chart of the study. ART: assisted reproductive technology; GDM: gestational diabetes mellitus; GHT: gestational hypertension; HELLP: hemolysis-elevated liver enzymes-low platelet; PE: preeclampsia; PGDM: pregestational diabetes; PPROM: preterm premature rupture of membranes.

Exclusion criteria were as follows: multiple pregnancies, chronic inflammatory, autoimmune, or infectious diseases; and maternal comorbidities including malignancies, cardiovascular disorders, or rheumatological conditions. Pregnancies complicated by active viral or bacterial infections, conception via assisted reproductive techniques, or inflammation-related obstetric complications—including intrahepatic cholestasis, premature rupture of membranes, or preeclampsia—were also excluded. Additionally, cases with major fetal anomalies detected on ultrasound or with inadequate imaging quality precluding reliable fetal thymus assessment were excluded. The flow chart including detailed information about the study cohort is shown in Figure 1.

The required sample size was determined a priori using a power calculation to detect a 10% difference in the fetal TTR between groups, with 80% statistical power at a two-sided significance level of 0.05, assuming a standard deviation of 0.05.

### 2.3. Diabetes Screening and Group Stratification

At the second-trimester visit (20–22 weeks), women at risk for PGDM who met early screening criteria underwent either random blood glucose testing or a one-step 75 g oral glucose tolerance test (OGTT). Screening was considered positive if fasting plasma glucose was ≥126 mg/dL, the 2 h OGTT value was ≥200 mg/dL, or a random plasma glucose was ≥200 mg/dL in the presence of classical hyperglycemia symptoms [30]. Women meeting these criteria were classified into the PGDM group.

The remaining participants underwent routine GDM screening at 24–28 weeks of gestation using the one-step 75 g OGTT. A diagnosis of GDM was established if at least one plasma glucose value exceeded the diagnostic thresholds: fasting > 92 mg/dL, 1 h > 180 mg/dL, or 2 h > 153 mg/dL [31]. Women diagnosed with GDM were subsequently stratified into two subgroups according to treatment modality: diet-controlled GDM and insulin-treated GDM. The control group consisted of healthy pregnant women with normal glucose tolerance who did not meet the diagnostic criteria for either PGDM or GDM.

### 2.4. Ultrasound Assessment of the Fetal Thymus

All participants underwent regular prenatal follow-up from the second trimester until delivery. Ultrasound examinations were performed using the Voluson E6 system (GE Medical Systems, Milwaukee, WI, USA) by a single experienced sonographer to ensure standardization and minimize interobserver variability. Measurements were obtained during periods of fetal quiescence to reduce motion-related bias.

To maintain methodological consistency, only TTR measurements obtained within the standardized gestational window of 30–32 weeks were included in the final analysis. The thymus was identified as a homogeneous structure in the anterior mediastinum on the three-vessel and trachea view, with concurrent visualization of the fetal sternum and vertebrae [32]. The TTR was assessed employing the methodology outlined by Chaoui et al., whereby the anteroposterior thymic diameter was determined from the posterior margin of the sternum to the anterior wall of the aortic arch, and the anteroposterior mediastinal diameter was measured from the posterior margin of the sternum to the anterior border of the thoracic vertebrae [17]. The TTR was calculated as the ratio of these diameters, with a value ≤0.39 considered indicative of a reduced fetal thymus size based on established norms [17,33].

### 2.5. Calculation of Hematological Biomarkers

Complete blood counts (CBCs), CRP, albumin and fibrinogen levels were obtained from all participants between 30 and 32 weeks of gestation via standard venipuncture. Samples were processed within two hours of collection and analyzed using a calibrated hematology analyzer (Sysmex XN 1000, Sysmex Corporation, Kobe, Japan), which employs impedance and optical fluorescence technology. The analyzer was regularly calibrated in accordance with the manufacturer’s recommendations, and internal quality control samples were analyzed daily to ensure measurement reliability.

Hematological parameters were recorded, and systemic inflammatory indices were calculated as follows: NLR = neutrophil count/lymphocyte count; MLR = monocyte count/lymphocyte count; PLR = platelet count/lymphocyte count; SII = (neutrophil count × platelet count)/lymphocyte count; SIRI = (neutrophil count × monocyte count)/lymphocyte count; AISI = (neutrophil count × platelet count × monocyte count)/lymphocyte count; CAR = CRP/albumin; and FAR = fibrinogen/albumin.

In addition to laboratory and ultrasound assessments, maternal demographic and obstetric characteristics were recorded, including maternal age, gravidity, parity, and pre-pregnancy body mass index (BMI). Neonatal outcomes were also recorded, including gestational age at delivery, birth weight, Apgar scores at 1 and 5 min, umbilical artery pH, and the requirement for neonatal intensive care unit (NICU) admission.

### 2.6. Statistical Analysis

Data analysis was conducted using SPSS version 26.0 (SPSS Inc., Chicago, IL, USA). Continuous variables were expressed as mean ± standard deviation (SD) or median with interquartile range (IQR), determined by their distributional properties as assessed via the Shapiro–Wilk test. Variables exhibiting normal distribution were compared using the independent *t*-test, whereas those with non-normal distribution were analyzed using the Mann–Whitney U test. For comparisons involving more than two groups, the Kruskal–Wallis test was employed, with post hoc pairwise comparisons conducted using the Mann–Whitney U test where statistically justified. Categorical variables were evaluated using the chi-squared test, with Bonferroni correction applied to account for multiple comparisons. Receiver operating characteristic (ROC) curve analysis was performed to assess the diagnostic performance of hematological and inflammatory biomarkers in detecting reduced fetal thymus size. Cut-off values, sensitivity, specificity, and the area under the curve (AUC) were derived, with the Youden index (J = sensitivity + specificity − 1) utilized to determine the optimal threshold. Statistical significance was defined as a two-sided *p*-value < 0.05.

## 3. Results

Of the 718 pregnant women who met the eligibility criteria and consented to participate, 186 were excluded from the final analysis. A total of 532 participants, including 166 with diabetes mellitus, were included in the final analysis. As shown in Table 1, women in the diabetic cohort had significantly higher mean maternal age, pre-pregnancy BMI, and lower gestational age at delivery compared with controls (all *p* < 0.001). Neonates born to diabetic mothers exhibited lower umbilical artery pH, lower Apgar scores at both 1 and 5 min, and higher rates of NICU admission (*p* = 0.003, *p* < 0.001, *p* < 0.001, and *p* < 0.001, respectively).

Comparative analyses of systemic inflammatory and hematological parameters among the study groups are presented in Table 2, comprising PGDM (*n* = 44), diet-controlled GDM (*n* = 73), insulin-treated GDM (*n* = 49), and normoglycemic controls (*n* = 366). The PGDM group exhibited significantly higher mean platelet volume (MPV), NLR, PLR, MLR, SIRI, and AISI values (*p* = 0.001, *p* = 0.002, *p* = 0.002, *p* < 0.001, *p* = 0.002, and *p* < 0.001, respectively). The insulin-treated GDM group demonstrated significantly higher fibrinogen, FAR, and CAR values compared with the other groups (all *p* < 0.001). In addition, albumin levels were significantly decreased in both the PGDM and insulin-treated GDM groups (*p* < 0.001). No statistically significant differences in platelet counts were observed among the groups (*p* = 0.054).

As shown in Table 3, sonographic TTR values were lowest in the PGDM group, followed by the insulin-treated GDM group (*p* < 0.001).

The diagnostic performance of systemic inflammatory biomarkers in predicting reduced fetal thymus size was further assessed using ROC curve analysis (Table 4). Among the evaluated indices, AISI and FAR demonstrated the highest predictive accuracy. For AISI, an optimal cut-off value of 640.3 yielded 82.3% sensitivity and 86.7% specificity, with an AUC of 0.891 (95% CI: 0.723–0.955, *p* < 0.001). The optimal cut-off for FAR was 0.114, providing 74.3% sensitivity and 88.7% specificity, with an AUC of 0.879 (95% CI: 0.686–0.919, *p* < 0.001).

## 4. Discussion

The present study evaluated the predictive value of maternal systemic inflammatory indices and hematological parameters across distinct diabetes subgroups in relation to fetal thymic size in the third trimester, yielding findings with potential clinical implications. The results demonstrate that maternal inflammatory burden varies according to glycemic status and exhibits a robust association with reduced fetal thymic dimensions. Both gestational and pregestational diabetes were associated with a significant reduction in thymic size compared to normoglycemic controls, with the most substantial decrease observed in the pregestational diabetes cohort.

Distinct biomarker patterns were evident across subgroups. Maternal serum levels of NLR, PLR, MLR, SIRI, AISI, and MPV were significantly elevated in the pregestational diabetes, whereas CAR, FAR, and fibrinogen were predominantly increased in the insulin-treated gestational diabetes. Conversely, albumin levels were consistently reduced in both the pregestational and insulin-treated gestational diabetes groups, likely reflecting a hepatic shift toward increased synthesis of acute-phase reactants, such as CRP and fibrinogen, as part of the systemic inflammatory response associated with impaired glycemic control. Several indices—including NLR, PLR, MLR, SII, SIRI, AISI, CAR, and FAR—emerged as significant predictors of reduced fetal thymic size, with AISI and FAR demonstrated the highest diagnostic performance. To our knowledge, this is the first prospective study to establish these associations, providing novel insights into the intricate interplay between maternal metabolic disturbances, systemic inflammation, and fetal immune development. Furthermore, it highlights the clinical utility of simple, widely accessible hematological biomarkers as non-invasive tools for identifying fetuses at risk of immune dysregulation.

Although significant progress has been made in perinatal medicine, the reliable prediction of postnatal immune health during fetal life remains challenging [34,35,36,37]. The increasing prevalence of chronic immune-mediated diseases, such as asthma, eczema, allergic rhinitis, and food allergies, represents a major public health concern, particularly given their earlier onset in childhood. Although genetic predisposition contributes to susceptibility, hereditary factors alone do not account for the sustained rise observed over recent decades [37]. Emerging evidence suggests that prenatal and perinatal factors—including maternal metabolic status, chronic low-grade inflammation, environmental exposures, and psychosocial stressors—play a decisive role in shaping fetal immune development [38,39,40,41,42,43]. The current findings support this paradigm by demonstrating a direct association between systemic inflammation in diabetic pregnancies and impaired fetal thymic growth, a key marker of disrupted immune ontogeny.

The thymus, as the primary lymphoid organ, serves as the central site of T-cell maturation, which is essential for establishing adaptive immunity. Prenatal perturbations in thymic development may impair T-cell differentiation and regulatory T-cell (Treg) function, predisposing offspring to long-term immune-mediated disorders [15]. Alterations in thymic size, such as hypoplasia or hyperplasia, have been documented across various pathological contexts and extensively studied in relation to fetal inflammatory response syndrome (FIRS), which is associated with intrauterine infection and inflammation [44,45]. Reduced thymic size has been consistently correlated with these inflammatory states, positioning it as a surrogate marker of compromised immune development [46,47]. Ultrasonographic assessment of fetal thymic size, particularly via TTR, may thus offer a valuable screening strategy to identify at-risk fetuses.

Previous studies have demonstrated that TTR remains stable throughout gestation and is unaffected by maternal BMI, with a mean value of approximately 0.44 in normal fetuses [17,33]. In the present study, the control group exhibited a mean TTR of 0.43, whereas significantly lower values were observed in the diabetes subgroups: 0.40 in diet-regulated GDM, 0.34 in insulin-treated GDM, and 0.29 in PGDM. These results underscore the potential of TTR as a robust marker of impaired fetal thymic development in diabetic pregnancies. Our results are consistent with the limited number of previous studies investigating the impact of maternal diabetes on fetal thymus size [46,47]. Dörnemann et al., in a case–control study involving 322 pregnant women, identified a significant association between maternal diabetes and decreased fetal thymus size [48]. However, in contrast to our findings, no significant differences were reported between the diabetes subgroups in their cohort, which may be attributable to the retrospective design and relatively limited sample size of the study. Conversely, Warncke et al. exclusively investigated autoimmune type 1 diabetes (T1D), excluding other forms of pregestational type 2 diabetes and gestational diabetes. They observed no reduction in fetal thymic size, potentially attributable to stringent metabolic control in patients with T1D during pregnancy [49].

Despite these insights, prenatal ultrasonographic thymic assessment has not been incorporated into routine obstetric care. The increasing burden of chronic immune-mediated diseases highlights the urgent need for alternative, accessible biomarkers as indirect indicators of fetal thymic development. Hematological indices derived from CBC parameters—including NLR, PLR, SII, SIRI, MLR, FAR, CAR, and AISI—are already widely utilized in clinical practice for risk stratification in inflammatory and metabolic disorders [27,50,51]. Their primary advantage lies in their cost-effectiveness and accessibility, as they are derived from standard blood tests that require no specialized equipment or advanced laboratory infrastructure. The present study demonstrates that these indices, particularly AISI and FAR, could complement ultrasonographic evaluation, providing a practical approach to identify fetuses at risk of immune dysregulation, especially in clinical settings where direct thymic imaging is constrained or impractical.

Increasing attention has been directed toward transplacental immune regulation, which has emerged as a critical area of research due to its decisive role in shaping fetal immune maturation through maternal–fetal immune interactions. Although the placenta typically separates maternal and fetal immune cells, growing evidence suggests a significant cross-talk between these immune systems [52,53,54,55]. Santner-Nanan et al. demonstrated an alignment of maternal and fetal cellular immunity, particularly in Tregs, likely mediated by interleukin-10 (IL-10) in healthy pregnancies [56]. Diemert et al. reported a negative correlation between Treg cells in cord blood and fetal thymus size [57], whereas Warncke et al. found no such correlations, except for a modest association with basophils in maternal peripheral blood [49]. These findings highlight the complexity of immune interactions and warrant further investigation to elucidate their impact on thymic development in diabetic pregnancies.

Maternal hyperglycemia is increasingly implicated as a key driver of subclinical inflammation, acting as a fetal stressor that contributes to the pathogenesis of FIRS [58,59,60,61]. Furthermore, chronic low-grade inflammation associated with maternal dysglycemia disrupts cytokine balance and alters hematological indices, potentially leading to reduced thymic size and maladaptive immune programming. By establishing evidence-based cut-off values for inflammatory biomarkers—such as AISI and FAR—in relation to fetal thymus size, this study offers a clinically actionable framework for predicting impaired thymic development. These findings highlight the potential for integrating such biomarkers into routine prenatal care to enhance the early identification of fetuses at risk of long-term immune dysregulation.

This study has several limitations that should be acknowledged. First, despite a relatively large, prospectively collected sample, the single-center design at a tertiary facility may limit the generalizability of the findings to populations with diverse demographic or clinical characteristics. Second, although this study focused on readily available inflammatory biomarkers, these markers provide indirect assessments of systemic inflammation and do not fully elucidate the complex cytokine signaling or cellular immune pathways involved in fetal immune development. Incorporating molecular biomarkers or immunophenotyping could provide deeper mechanistic insights. Third, the absence of neonatal and long-term postnatal immune outcome data precludes definitive conclusions about the predictive efficacy of these biomarkers for subsequent immune function. Nevertheless, the strengths of this study include its prospective design, relatively large sample size, standardized sonographic assessment of the thymus, and comprehensive evaluation of widely available hematological indices.

Future research should seek to validate these findings in larger, multicenter cohorts encompassing diverse populations. Integrating inflammatory biomarkers with advanced imaging modalities and molecular immune profiling may improve predictive accuracy and enhance the understanding of mechanisms linking maternal inflammation, metabolic status, and fetal thymic development. Longitudinal studies extending into the postnatal period are particularly warranted to determine whether observed reductions in thymus size correlate with clinically significant alterations in immune competence or increased susceptibility to immune-mediated diseases in childhood. Ultimately, such investigations could facilitate the development of cost-effective, non-invasive screening strategies that integrate ultrasonographic and hematological markers to identify high-risk pregnancies and guide early preventive interventions.

## 5. Conclusions

Our findings demonstrate that maternal inflammatory burden varies according to glycemic status during pregnancy and correlates with quantifiable reductions in fetal thymus size. Among the evaluated biomarkers, AISI and FAR exhibited the highest predictive performance. The increasing prevalence of chronic immune-mediated diseases, coupled with their earlier onset in childhood, highlights the urgent need for reliable clinical markers of fetal immune development. Such markers could serve as valuable tools for the early identification of offspring at increased risk of postnatal autoimmune disorders, thereby facilitating timely preventive or therapeutic interventions to mitigate long-term immune dysregulation, particularly in clinical settings where direct thymus imaging is limited or impractical.

## Figures and Tables

**Table 1:** Maternal demographic and perinatal outcomes of the study population.

	Diabetes Group (*n* = 166)	Control Group (*n* = 366)	*p* Value *
Age (years)	31.5 ± 4.30	28 ± 5.12	<0.001 ^a^
Gravidity	2.48 ± 1.24	2.34 ± 1.18	0.326 ^b^
Parity	1.16 ± 1.32	1.02 ± 0.81	0.642 ^a^
Pre-pregnancy BMI (kg/m^2^)	30.5 ± 2.25	26.9 ± 1.24	<0.001 ^a^
Gestational age at delivery (weeks)	38.2 ± 1.4	39.3 ± 1.7	<0.001 ^a^
Birth weight (g)	3345 ± 654.2	3265 ± 458.5	0.076 ^a^
1 min Apgar score	8 (5–9)	9 (7–9)	<0.001 ^b^
5 min Apgar score	8 (7–10)	9 (8–10)	<0.001 ^b^
NICU admission	35 (21%)	10 (3%)	<0.001 ^c^
Umbilical artery pH	7.36 ± 0.02	7.39 ± 0.08	0.003 ^a^

Abbreviations: BMI: body mass index; NICU: neonatal intensive care unit. Data presented as mean ± standard deviation; median (minimum-maximum) or as number (percentage). * *p* values calculated using: ^a^ Independent *T* test; ^b^ Mann–Whitney U test; ^c^ chi-squared; *p* < 0.05 was considered statistically significant.

**Table 2:** Comparative analysis of systemic inflammatory indices and hematological parameters among the study groups.

	PGDM ^a^ (*n* = 44)	GDM (Diet-Controlled) ^b^ (*n* = 73)	GDM (Insulin-Treated) ^c^ (*n* = 49)	Control ^d^ (*n* = 366)	*p* Value	*p* Value *
WBC (10^9^/L) (mean ± SD)	11.67 ± 2.49	10.75 ± 6.71	11.92 ± 1.25	10.2 ± 3.40	<0.001	a = c > b > d
NEU (10^9^/L) (mean ± SD)	8.94 ± 4.31	7.98 ± 2.14	8.73 ± 4.35	7.65 ± 1.82	0.002	a > c > b > d
LNF (10^9^/L) (mean ± SD)	1.48 ± 0.44	1.88 ± 0.25	1.54 ± 0.29	1.91 ± 0.14	0.048	d = b > c = a
MON (10^9^/L) (mean ± SD)	0.65 ± 0.31	0.51 ± 0.47	0.62 ± 0.17	0.49 ± 0.31	0.009	a = c > b = d
PLT (10^9^/L) (mean ± SD)	225.26 ± 43.38	230.37 ± 56.81	227.17 ± 31.37	232.17 ± 18.21	0.054	b = c = d = a
MPV (fL) (mean ± SD)	9.78 ± 0.56	7.98 ± 0.73	8.62 ± 0.21	7.55 ± 0.97	0.001	a > c > b >d
NLR (mean ± SD)	5.32 ± 2.31	3.25 ± 1.64	3.98 ± 5.21	2.78 ± 1.93	0.002	a >c > b > d
PLR (mean ± SD)	168.22 ± 29.41	132.18 ± 64.19	152.54 ± 46.57	118.74 ± 38.73	0.002	a > c > b > d
MLR (mean ± SD)	0.439 ± 0.339	0.271 ± 0.286	0.403 ± 0.187	0.257 ± 0.182	<0.001	a > c > b = d
SII (mean ± SD)	1328.7 ± 901.4	985.43 ± 675.7	1245.25 ± 312.9	868.32 ± 585.3	<0.001	a = c > b > d
SIRI (mean ± SD)	3.58 ± 1.18	2.01 ± 1.11	3.18 ± 1.32	1.61 ± 1.02	0.002	a > c > b > d
AISI median (IQR)	861 (125)	499 (93)	798 (141)	456 (136)	<0.001	a > c > b > d
Albumin (g/L) (mean ± SD)	34 ± 3.31	35 ± 3.36	33 ± 3.32	38 ± 3.39	<0.001	d > b > a = c
Fibrinogen (g/L) (mean ± SD)	4.24 ± 1.2	4.10 ± 1.2	4.35 ± 1.3	3.78 ± 1.1	<0.001	c > a > b > d
CRP (mg/L) (mean ± SD)	8.4 ± 3.71	7.5 ± 3.11	8.9 ± 4.23	6.3 ± 2.81	<0.001	a = c > b > d
FAR (mean ± SD)	0.125 ± 0.03	0.117 ± 0.02	0.132 ± 0.03	0.100 ± 0.02	<0.001	c > a > b > d
CAR (mean ± SD)	0.247 ± 0.06	0.214 ± 0.05	0.270 ± 0.06	0.166 ± 0.05	<0.001	c > a > b > d

Abbreviations: WBC: white blood cells; NEU: neutrophil; LNF: lymphocyte; MON: monocyte; PLT: platelet; MPV: mean platelet volume; NLR: neutrophil-to-lymphocyte ratio; PLR: platelet-to-lymphocyte ratio; MLR: monocytes-to-lymphocytes; SII: systemic immune-inflammation index (neutrophils × platelets)/lymphocytes; SIRI: systemic inflammatory response index (neutrophils × monocytes)/lymphocytes; AISI: aggregate index of systemic inflammation (neutrophils × platelets × monocytes)/lymphocytes; CRP: C-reactive protein; CAR: CRP-to-albumin; FAR: fibrinogen-to-albumin; PGDM: pregestational diabetes; GDM: diabetes mellitus; SD: standard deviation; NS: not significant; Kruskal–Wallis * (Mann–Whitney U test) statistics; *p* < 0.05 compared with the control groups. ^a^: PGDM; ^b^: GDM (diet-controlled); ^c^: GDM (insulin-treated); ^d^: control.

**Table 3:** Comparision of the fetal thymus size among the study cohort.

	PGDM ^a^ (*n* = 44)	GDM (Diet-Controlled) ^b^ (*n* = 73)	GDM (Insulin-Treated) ^c^ (*n* = 49)	Control ^d^ (*n* = 366)	*p* Value	*p* Value *
Gestational age at ultrasound (weeks) median (min-max)	30.5 (29.0–31.6)	30.6 (29.1–32.0)	30.52 (29.2–31.5)	30.56 (29.0–31.6)	0.17	NS
Thymic-thoracic ratio (TTR) median (min-max)	0.290 (0.24–0.441)	0.400 (0.29–0.431)	0.340 (0.250–0.421)	0.435 (0.389–0.472)	<0.001	d > b > c > a

Abbreviations: PGDM: pregestational diabetes mellitus; GDM: diabetes mellitus; NS: not significant; Kruskal–Wallis * (pairwise comparisions with Bonferroni correction) statistics; *p* < 0.05 compared with the control groups. ^a^: PGDM; ^b^: GDM (diet-controlled); ^c^: GDM (insulin-treated); ^d^: control.

**Table 4:** Receiver operating characteristic (ROC) analysis of systemic inflammatory biomarkers for predicting reduced fetal thymus size.

Analyzed Marker	Cut-Off Value	Sensitivity	Specificity	AUC	95% CI	*p* Value
AISI	640.3	0.823	0.867	0.891	0.723–0.955	<0.001
CAR	0.239	0.646	0.882	0.775	0.614–0.925	0.001
SII	1085.23	0.632	0.764	0.723	0.631–0.913	<0.001
SIRI	2.388	0.642	0.824	0.786	0.591–0.887	<0.001
PLR	132.764	0.728	0.748	0.821	0.603–0.891	0.001
MLR	0.418	0.636	0.753	0.754	0.596–0.917	<0.001
FAR	0.114	0.7432	0.887	0.879	0.686–0.919	<0.001
NLR	4.18	0.783	0.875	0.793	0.613–0.931	<0.001

Abbreviations: NLR: neutrophil-to-lymphocyte ratio; PLR: platelet-to-lymphocyte ratio; MLR: monocytes-to-lymphocytes; SII: systemic immune-inflammation index (neutrophils × platelets)/lymphocytes; SIRI: systemic inflammatory response index (neutrophils × monocytes)/lymphocytes; AISI: aggregate index of systemic inflammation (neutrophils × platelets × monocytes)/lymphocytes; CRP: C-reactive protein; CAR: CRP-to-albumin; FAR: fibrinogen-to-albumin; AUC: area under the curve; CI: confidence interval; *p* < 0.05 compared with the control groups.

## Data Availability

The data supporting the findings of this study are available from the corresponding author (GB) upon reasonable request.
